# Degraded inferior colliculus responses to complex sounds in prenatally exposed VPA rats

**DOI:** 10.1186/s11689-023-09514-9

**Published:** 2024-01-02

**Authors:** Yuko Tamaoki, Varun Pasapula, Collin Chandler, Michael S. Borland, Olayinka I. Olajubutu, Liza S. Tharakan, Crystal T. Engineer

**Affiliations:** 1 School of Behavioral and Brain Sciences, The University of Texas at Dallas, 800 West Campbell Road BSB11, Richardson, TX 75080 USA; 2 The University of Texas at Dallas, Texas Biomedical Device Center, 800 West Campbell Road BSB11, Richardson, TX 75080 USA

**Keywords:** Autism, Auditory processing, Inferior colliculus, Valproic acid

## Abstract

**Background:**

Individuals with autism spectrum disorders (ASD) often exhibit altered sensory processing and deficits in language development. Prenatal exposure to valproic acid (VPA) increases the risk for ASD and impairs both receptive and expressive language. Like individuals with ASD, rodents prenatally exposed to VPA exhibit degraded auditory cortical processing and abnormal neural activity to sounds. Disrupted neuronal morphology has been documented in earlier processing areas of the auditory pathway in VPA-exposed rodents, but there are no studies documenting early auditory pathway physiology. Therefore, the objective of this study is to characterize inferior colliculus (IC) responses to different sounds in rats prenatally exposed to VPA compared to saline-exposed rats.

**Methods:**

In vivo extracellular multiunit recordings from the inferior colliculus were collected in response to tones, speech sounds, and noise burst trains.

**Results:**

Our results indicate that the overall response to speech sounds was degraded in VPA-exposed rats compared to saline-exposed controls, but responses to tones and noise burst trains were unaltered.

**Conclusions:**

These results are consistent with observations in individuals with autism that neural responses to complex sounds, like speech, are often altered, and lays the foundation for future studies of potential therapeutics to improve auditory processing in the VPA rat model of ASD.

**Supplementary Information:**

The online version contains supplementary material available at 10.1186/s11689-023-09514-9.

## Introduction

Individuals with autism spectrum disorders (ASD) often exhibit altered sensory processing and deficits in language development. This manifests as a range of auditory processing challenges across individuals, from a heightened sensitivity to sound to a diminished sensitivity to sound. These perceptual changes are accompanied by alterations in the neural response to sound across the auditory pathway [1, 2]. For example, auditory cortex responses to vowel sounds are significantly delayed in verbal children with ASD compared to typically developing children, even further delayed in language-impaired children with ASD, and profoundly delayed in minimally verbal and nonverbal children with ASD [3]. Children with ASD benefit from traditional rehabilitation therapies, but many children undergo these time-consuming expensive therapies and still experience deficits. The development of new techniques to enhance auditory processing in individuals with neurodevelopmental disorders is necessary.

Children who are prenatally exposed to the anticonvulsant medication valproic acid (VPA) have an increased risk for ASD, learning impairments, and developmental delays [4]. Prenatal exposure to VPA can result in ASD symptoms including altered sensory processing and deficits in language development [5–8]. Children prenatally exposed to VPA have impaired receptive and expressive language that is dose-dependent: children who were prenatally exposed to larger VPA doses have worse language abilities than children who were exposed to smaller doses [9].

Like humans, rodents prenatally exposed to VPA exhibit degraded auditory processing and abnormal cortical neural activity to sounds. VPA-exposed rats are impaired at discriminating between sounds differing in the initial consonant (“dad” vs. “bad”) compared to saline-exposed control rats [10]. In the auditory cortex, responses are weaker, delayed, and have disorganized tonotopy in VPA-exposed rodents [1, 11, 12]. In subcortical structures, fewer neurons and disrupted neuronal morphology have been documented in the superior olivary complex, lateral lemniscus, and inferior colliculus in VPA-exposed animals [13–16]. Additionally, prenatal VPA exposure results in more cFos labeling in the cochlear nucleus, superior olivary complex, and central nucleus of the inferior colliculus (CNIC) in response to pure tones, and reduced projections to the CNIC from the superior olivary complex, cochlear nucleus, and medial geniculate nucleus of the thalamus [13, 17, 18]. These neural changes are likely to cause disruption in the subcortical processing of sounds, but the existence and nature of any subcortical changes in physiology have not yet been characterized. Therefore, in this study, we tested the hypothesis that prenatal exposure to VPA degrades neural responses to sounds in a subcortical region of auditory processing, the central nucleus of the inferior colliculus (CNIC).

## Methods

Inferior colliculus (IC) responses were recorded from two groups of rats: (1) saline-exposed rats (*n* = 19), and (2) VPA-exposed rats (*n* = 18). The University of Texas at Dallas Institutional Animal Care and Use Committee approved all surgical protocols and recording procedures.

### Subjects

Male and female Sprague Dawley rats were obtained from Charles River Laboratory (Wilmington, MA) and mated. Pregnancy was determined by the presence of a vaginal plug. On embryonic day 12.5 of pregnancy, the female rats received a single intraperitoneal injection of either 600 mg/kg of valproic acid (VPA) dissolved in 0.9% saline or 0.9% saline alone, as in previous studies [10, 11, 19–21]. Thirty-seven male and female animals from ten litters were used for this study. Of the thirty-seven rats, 9 females and 10 males were used in the saline group, and 8 females and 10 males were used in the VPA group.

### Electrophysiology recording

**Fig. 1:**
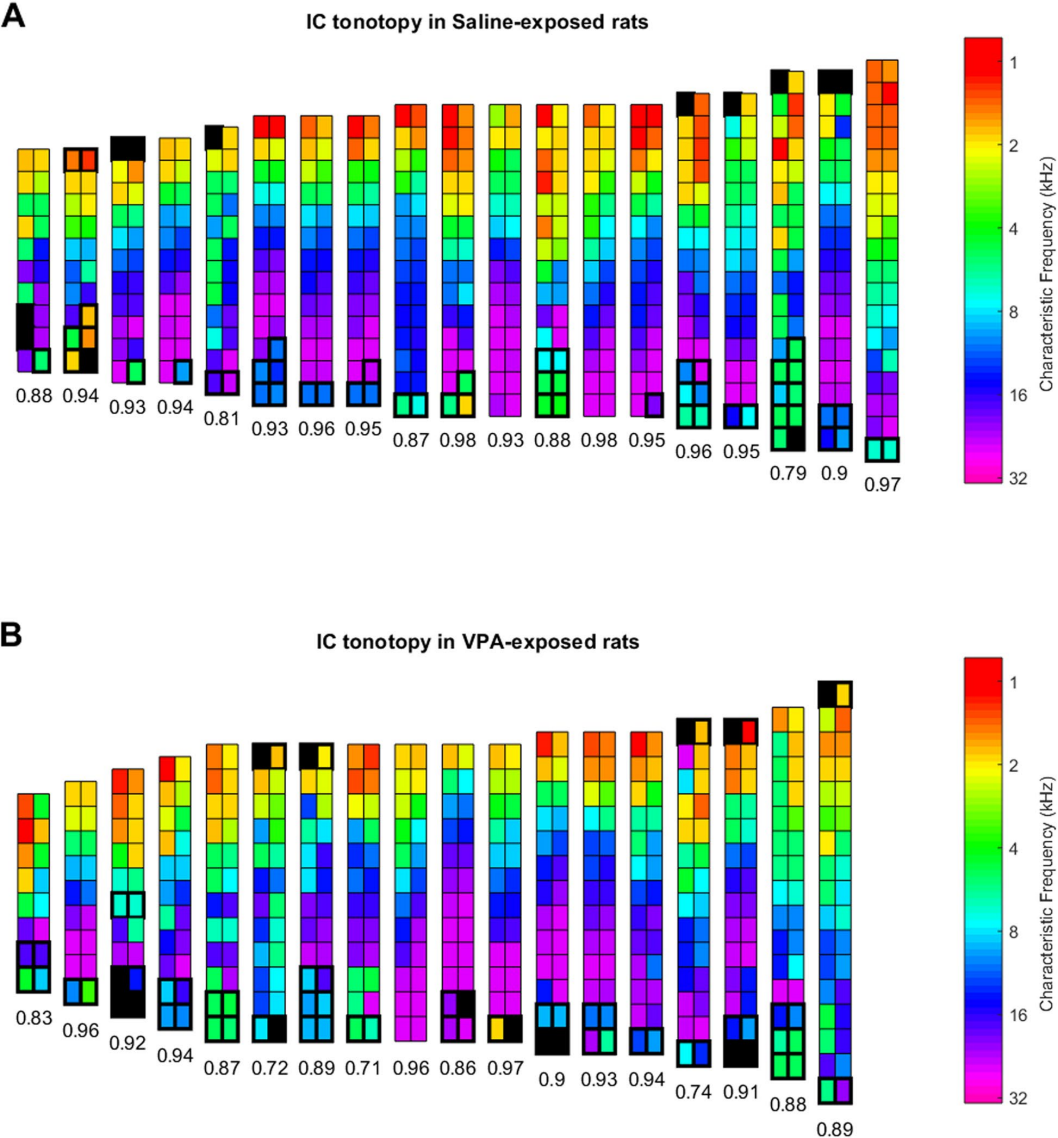
IC tonotopy in **A** saline-exposed rats, and **B** VPA-exposed rats. Each vertical column indicates the recording sites obtained in a single rat. Color indicates the characteristic frequency of each recording site. Filled black rectangles indicate recording sites without a clear characteristic frequency. The vertical distance between each recording site was 200 microns, and the horizontal distance between each recording site was 250 microns. Sites outlined in thick black lines were identified as other IC areas or were poorly tuned, and these sites were excluded from the analysis. Numbers beneath each column indicate the *R* value obtained between the recording location and the characteristic frequency, in octaves

Multi-unit inferior colliculus responses were recorded from 490 sites in the saline-exposed control group, and 415 sites in the VPA-exposed group. All recordings were obtained in adult rats over 90 days of age. The rats were anesthetized with pentobarbital (50 mg/kg), and booster pentobarbital (8 mg/kg) was administered as needed. To ease the animal’s breathing, a tracheotomy was performed to directly deliver humidified air. Following the tracheotomy, a hole was drilled in the skull 9 mm posterior and 1.5 mm lateral to bregma over the right inferior colliculus. Two Parylene-coated tungsten microelectrodes spaced 250 µm apart (1–2 MΩ, FHC) were lowered perpendicular to the cortical surface to 1000 microns below the pial surface and recordings were made at 200-micron intervals along the dorsal–ventral axis until a depth of approximately 4000–5000 microns. To ensure that the recordings were obtained from the central nucleus of the IC (CNIC), tonotopic organization and response latency were quantified, consistent with the methods used in previous studies [22–27]. From this perpendicular angle, the CNIC has a very clear tonotopic organization in which the characteristic frequency progresses from 1 to 32 kHz in an orderly manner. In areas outside the CNIC, such as the dorsal cortex and the external cortex of the IC, the characteristic frequencies obtained did not span the entire five-octave frequency range of our tone-recording stimuli in an orderly manner. We recorded one complete pass spanning the entire tonotopic axis of the CNIC in each rat. A total of 453 CNIC sites were obtained in the saline-exposed control group, and 374 CNIC sites were obtained in the VPA-exposed group. The tonotopic axis of every animal from this study is plotted in Fig. 1, with the accompanying Pearson correlation coefficient between the recording location and the characteristic frequency in octaves (log2 of the characteristic frequency, Fig. 1).

At each 200-micron recording interval, auditory stimuli were presented through a Tucker Davis Technologies (TDT) MF-1 speaker that was placed 10 cm away from the left ear. The stimulus set at each recording site consisted of 1296 tones of different frequency and intensity combinations from 1 to 32 kHz in 0.0625 octave steps and 0 to 75 dB SPL in 5 dB steps that were randomly interleaved. Additionally, 20 repeats of each of 15 randomly interleaved speech sounds (consonant–vowel-consonant) that differed by the initial consonant or the vowel were presented (‘chad’, ‘dad’, ‘deed’, ‘dood’, ‘fad’, ‘gad’, ‘had’, ‘jad’, ‘sad’, ‘shad’, ‘tad’). All speech sounds were presented so that the loudest 100 ms of the vowel portion of the sound was 60 dB. Additionally, the sounds ‘dad’ and ‘shad’ were also presented at 45 dB and 75 dB. Finally, 20 repeats of a noise burst train consisting of 6 25-ms noise bursts at a rate of 10 Hz were presented.

### Statistical analysis

All data were analyzed using MATLAB software, SPSS version 27, and GraphPad Prism Version 10.1.0. The response strength evoked by speech sounds was calculated by taking the average number of driven spikes during the entire 400 ms duration of the sound, during the first 40 ms onset response to the consonant portion of the sound, and during the 300 ms response to the vowel portion of the sound. The consonants were subdivided based on differences in manner of articulation (stop consonants, affricates, and fricatives) [28]. Onset latency was determined as the first spike latency after the presentation of each sound, and peak latency was defined as the maximum firing rate latency [11, 29].

The nearest neighbor classifier was used to determine the neural discrimination accuracy between pairs of sounds by utilizing spike timing from single-trial responses to each of the speech stimuli presented [11, 29]. This classifier determined the similarity of single-trial responses to peri-stimulus time histogram (PSTH) templates generated from the remaining 19 repeats of the IC response that was evoked by each sound. The nearest-neighbor classifier identifies the sound most likely to have generated the single trial response under consideration using Euclidean distance. The onset response for all speech sounds was the initial 40 ms response to the consonant portion of the sound, binned using 1 ms precision. The consonant pairs included all speech sounds that differed in the initial consonant, with a particular focus on consonant pairs differing within the manner of articulation categories. For the vowel response, the classifier was provided with the vowel portion of the response, from 140 to 440 ms, using a single 300 bin [22]. The vowel pairs were ‘Dad-Deed’, ‘Dad-Dood’, and ‘Deed-Dood’. Classifier accuracy was quantified as the percentage of neural responses that were accurately assigned to the sound that generated the response.

Responses to tones at each IC recording site were also quantified. Receptive field properties (characteristic frequency, response threshold, bandwidth, response latencies, and spontaneous firing) in the inferior colliculus were calculated [24, 30]. Firing rate and vector strength were quantified for the responses to the noise burst trains. The firing rate from all 6 bursts was quantified by taking the average number of driven spikes. Vector strength was used to quantify the synchrony of the noise bursts [31, 32]. For all analyses, normal distributions were determined using the Lilliefors test for normality. When the population was normally distributed, a generalized linear mixed model (GLMM) with Bonferroni correction was used when multiple recording sites were nested within individual rats, a two-way ANOVA was used when a single value was obtained for each rat to compare group responses, and a Welch’s *t-*test was used to compare the tonotopic axis length of CNIC between the two groups. For the GLMM, the experimental group (VPA vs. saline) was evaluated as a fixed factor, and rat was evaluated as a random factor. Otherwise, non-parametric Mann–Whitney *U* statistics were used to compare group inferior colliculus responses to tones, speech, and noise bursts [33]. Ninety-five percent bootstrap confidence intervals of the median were computed using 50,000 samples.

## Results

### Prenatal exposure to VPA decreases CNIC tonotopic axis length

Congenital defects including smaller brain volumes are documented in prenatally exposed VPA animals [34, 35]. In our study, VPA-exposed rats had a significantly decreased CNIC tonotopic axis length compared to saline-exposed control rats (saline (mean ± SEM) = 2453 ± 98.93 microns; VPA (mean ± SEM) = 2067 ± 101 microns; Welch’s *t*-test, *t*(34.92) = 2.730, *p* = 0.0098, Fig. 1). This finding is consistent with previous studies that have documented that prenatal exposure to VPA alters the size of the auditory midbrain [34, 35].

### IC responses to speech sounds are altered in VPA-exposed rats

**Fig. 2:**
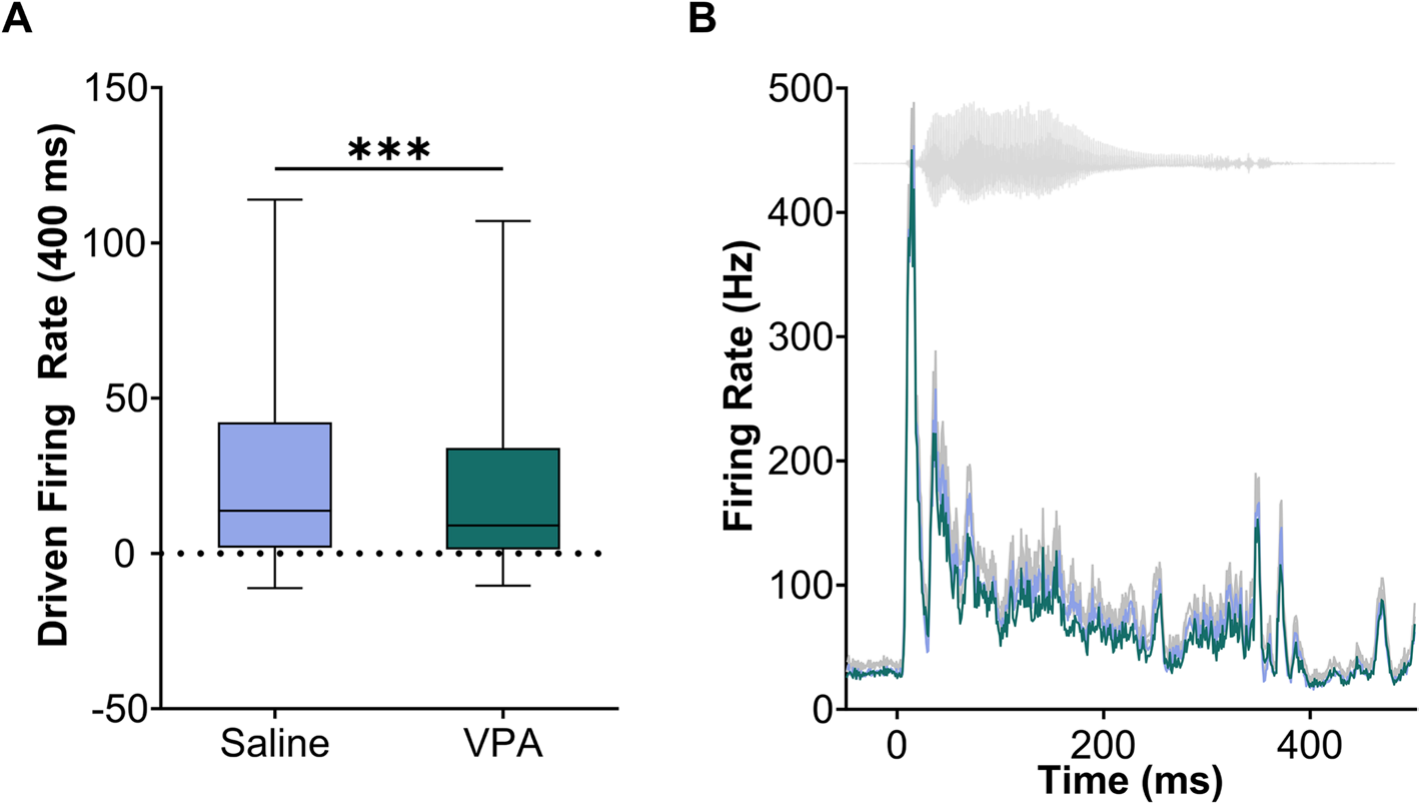
The IC response strength to speech sounds is weaker in VPA-exposed rats compared to saline-exposed rats. **A** Box and whisker plot showing the number of driven spikes evoked at each IC recording site for each speech sound. The driven rate was quantified using the 400 ms duration of the response. The black line indicates the median, and the error bars indicate the 95% confidence interval. The asterisks indicate experimental groups that are statistically significant from each other using a Mann–Whitney U test. The symbols represent the following: ***p < 0.001. **B** Post-stimulus time histogram showing the IC response to the sound ‘dad’ presented at 60 dB. The sound waveform is plotted behind the PSTH in gray

Delayed and weaker responses to sounds are commonly observed in both children and animal models of autism [1, 36–38]. VPA-exposed rats have also shown abnormal neural activity and degraded auditory cortical processing [10–12]. In our current study, we aimed to determine whether prenatal exposure to VPA could alter auditory responses in the IC. To do so, we first compared the IC responses to speech sounds between VPA-exposed rats and saline-exposed rats. Consistent with previous research [37, 39], we found that VPA-exposed rats had significantly weaker IC-driven responses to speech sounds than saline-exposed rats (Mann–Whitney *U*, *p* < 0.0001, Fig. 2a, b, Additional files 1 and 2).

**Fig. 3:**
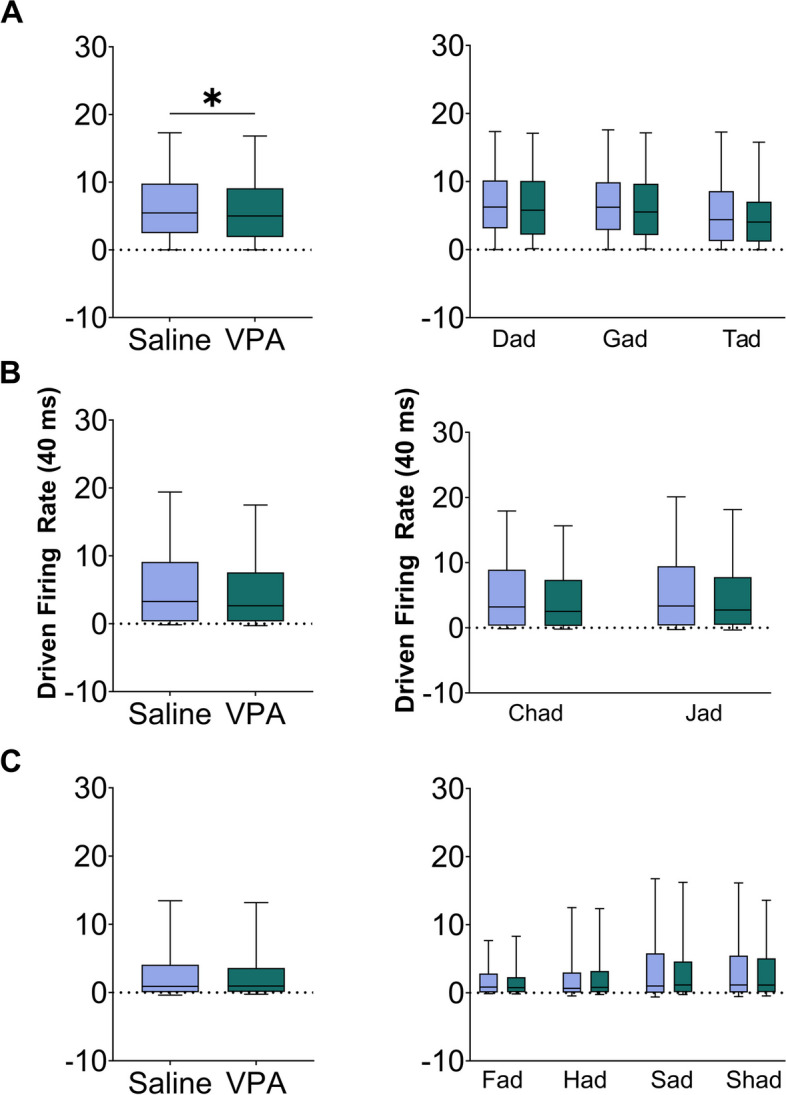
The IC response strength to the consonant portion of the speech sounds is altered in VPA-exposed rats compared to saline-exposed rats. **A** Left, box and whisker plots showing the number of driven spikes evoked at each IC recording site for the consonant portion of each stop consonant sound. The driven rate was quantified using the 40 ms response to the consonant portion of the sounds. The black line indicates the median, and the error bars indicate the 95% confidence interval. The asterisk indicates experimental groups that are statistically significant from each other using a Mann–Whitney *U* test. Right, box and whisker plots depicting the average group response strength evoked by the consonants ‘d’, ‘g’, and ‘t’ presented at 60 dB. **B** Left, box and whisker plots showing the number of driven spikes evoked at each IC recording site for the consonant portion of each affricate sound. Right, box and whisker plots depicting the average group response strength evoked by the consonants ‘ch’, and ‘j’ presented at 60 dB. **C** Left, box and whisker plots showing the number of driven spikes evoked at each IC recording site for the consonant portion of each fricative sound. The driven rate was quantified using the 40 ms response to the consonant portion of the sounds. Right, box and whisker plots depicting the average group response strength evoked by the consonants ‘f’, ‘h’, ‘s’, and ‘sh’ presented at 60 dB

The speech sounds were presented as consonant–vowel-consonant words and varied either in the initial consonant or the vowel portion of the word (i.e., ‘Dad’ vs ‘Gad’ or ‘Dad’ vs ‘Deed’). To determine whether VPA-exposed rats had deficits in specific portions of the sound, we compared responses between groups to both the consonant portion of the sounds and the vowel portion of the sounds. For consonant sounds, we first examined the IC response to stop consonants (‘d’, ‘g’, ‘t’), that are known to have rapid spectrotemporal transitions. There was a significant difference between groups in the response strength to the consonant portion of the sounds for stop consonants (Mann–Whitney *U*, *p* = 0.044, Fig. 3a, Additional file 3a). We next examined the IC response to affricate sounds (‘ch’ and ‘j’), which transition from stop consonant-like acoustics to fricative-like sounds, with a more rapid fricative portion of the sound compared to a pure fricative [28]. No significant difference between groups in the response strength to the consonant portion of the sound for affricate sounds was observed (Mann–Whitney *U*, *p* = 0.062, Fig. 3b, Additional file 3b). Finally, we examined the IC response to fricative sounds (‘f’, ‘h’, ‘s’, and ‘sh’), which involve a partial obstruction of the vocal tract. Similar to affricates, there was no significant alterations between groups in the response strength to the consonant portion of the sounds for fricative sounds (Mann–Whitney *U*, *p* = 0.399, Fig. 3c, Additional file 3c). These results indicate that the spectrotemporal acoustics of the consonant are an important factor in the IC response strength to consonant sounds.

**Fig. 4:**
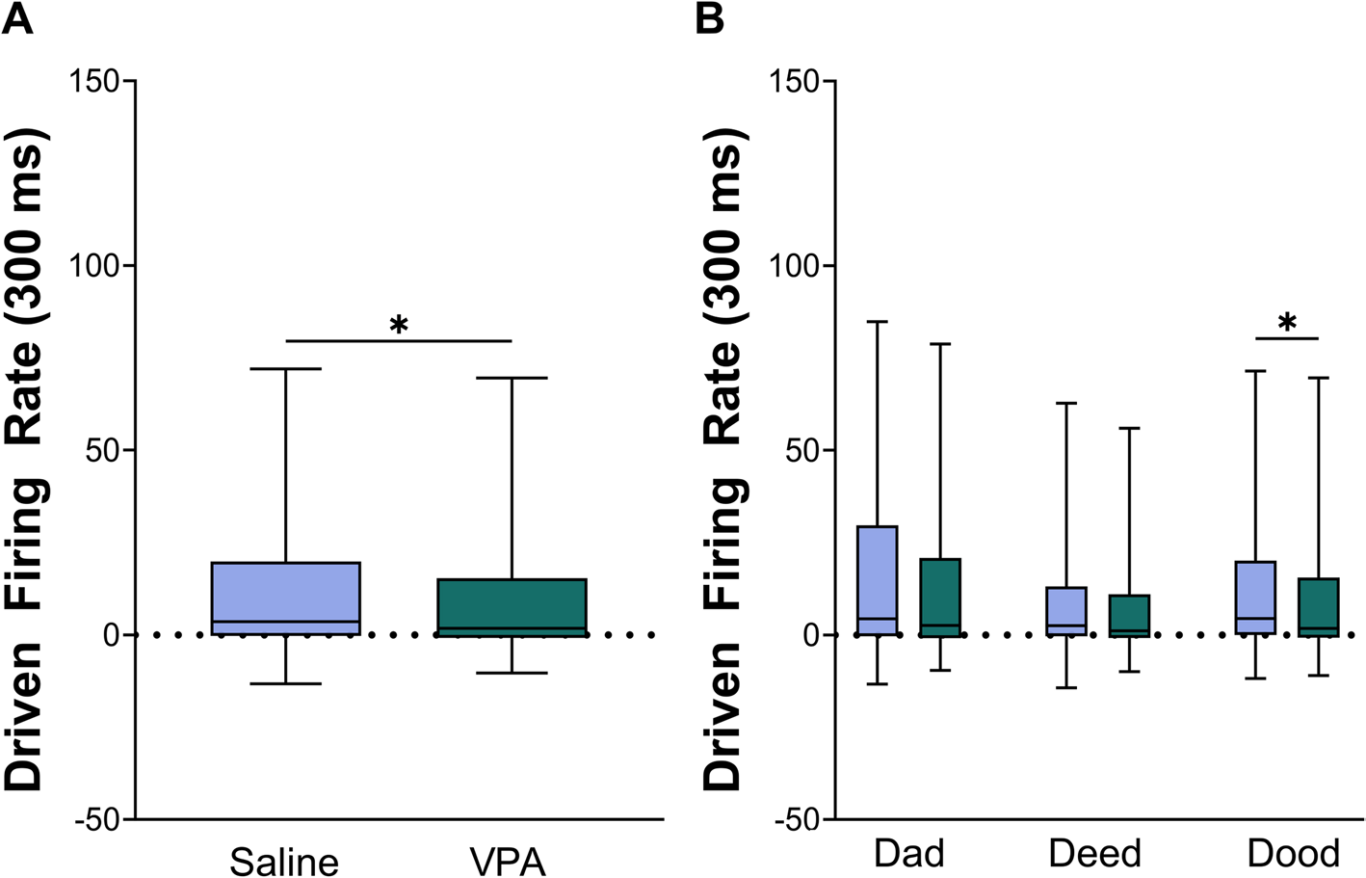
The IC response strength to the vowel portion of the speech sounds is altered in VPA-exposed rats compared to saline-exposed rats. **A** Box and whisker plots showing the number of driven spikes evoked at each IC recording site for the vowel portion of each speech sound. The driven rate was quantified using the 300 ms response to the vowel portion of the sounds. The black line indicates the median, and error bars indicate the 95% confidence interval. The asterisk indicates experimental groups that are statistically significant from each other using a Mann–Whitney *U* test. **B** Box and whisker plots depicting the average group response strength evoked by the vowels ‘a’, ‘ee’, and ‘oo’ presented at 60 dB

Similarly, the response strength to the vowel portion of the sounds was significantly altered, with VPA-exposed rats responding significantly weaker to the vowel portion of the sound compared to saline-exposed rats (Mann–Whitney *U*, *p* < 0.0001, Fig. 4, Additional file 4). Responses to both the consonant onset and the sustained vowel portions of the sound were significantly weaker in VPA-exposed rats compared to saline-exposed rats.

**Fig. 5:**
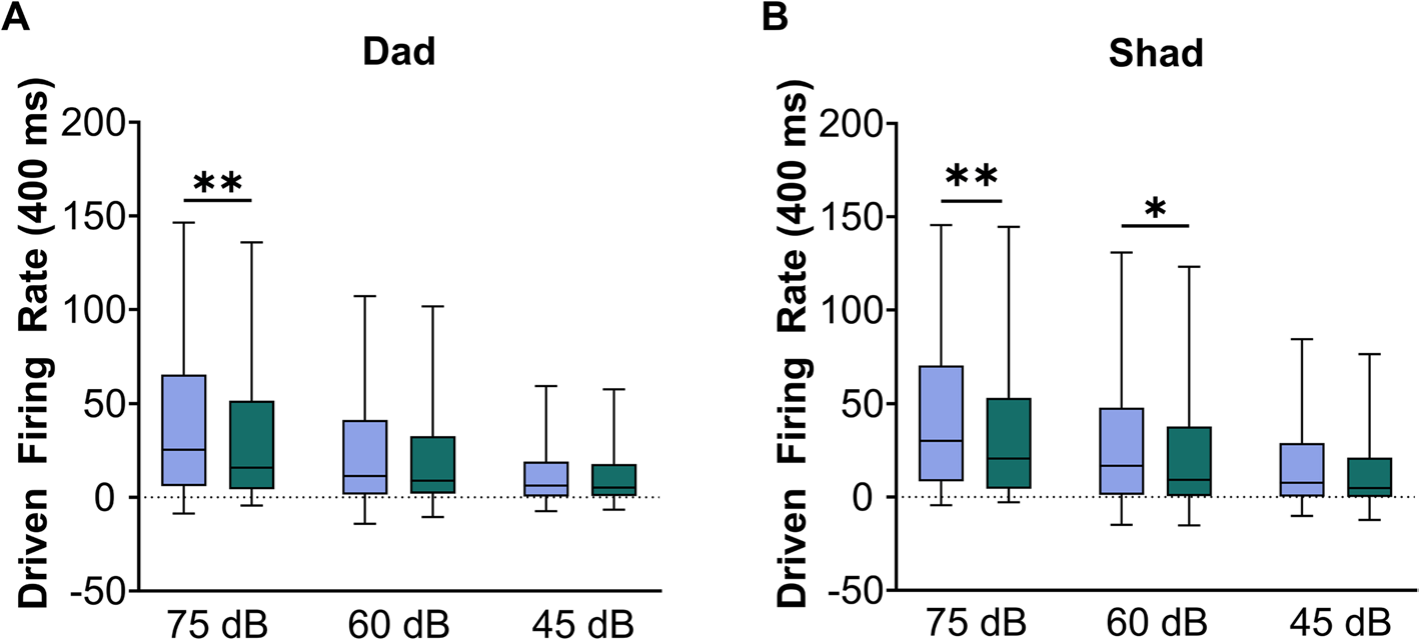
The IC response strength to speech sounds is altered over multiple sound intensities in VPA-exposed rats compared to saline-exposed rats. **A** Box and whisker plots depicting the average group response strength evoked by the sound ‘dad’ presented at 75, 60, and 45 dB. The driven rate was quantified using the 400 ms response to the sounds. The black line indicates the median, and the error bars indicate the 95% confidence interval. The asterisk indicates experimental groups that are statistically significant from each other using a Mann–Whitney *U* test. All asterisks are represented in APA style: **p < 0.005, *p < 0.05. **B** Box and whisker plots depicting the average group response strength evoked by the sound ‘shad’ presented at 75, 60, and 45 dB

In addition to presenting sounds differing in consonant or vowel, the sounds ‘Dad’ and ‘Shad’ were presented at three different intensities (75, 60, 45 dB). VPA-exposed rats had a significantly weaker response strength evoked by ‘dad’ presented at multiple sound intensities compared to the saline-exposed rats (Mann–Whitney *U*, *p* = 0.017, Fig. 5a, Additional file 5a). The sound ‘dad’ presented at 75 dB showed the greatest difference in response strength between the groups (Mann–Whitney* U*, *p* = 0.008). VPA-exposed rats also had a significantly weaker response strength evoked by ‘shad’ presented at multiple sound intensities compared to the saline-exposed rats (Mann–Whitney *U*, *p* < 0.0001, Fig. 5b, Additional file 5b). The sound ‘shad’ presented at 60 and 75 dB showed the greatest difference in response strength between the groups (Mann–Whitney *U*, 60 dB: *p* = 0.01; 75 dB: *p* = 0.008). These results suggest that VPA-exposed rats have a greater response strength deficit to louder sounds compared to quieter sounds.

**Fig. 6:**
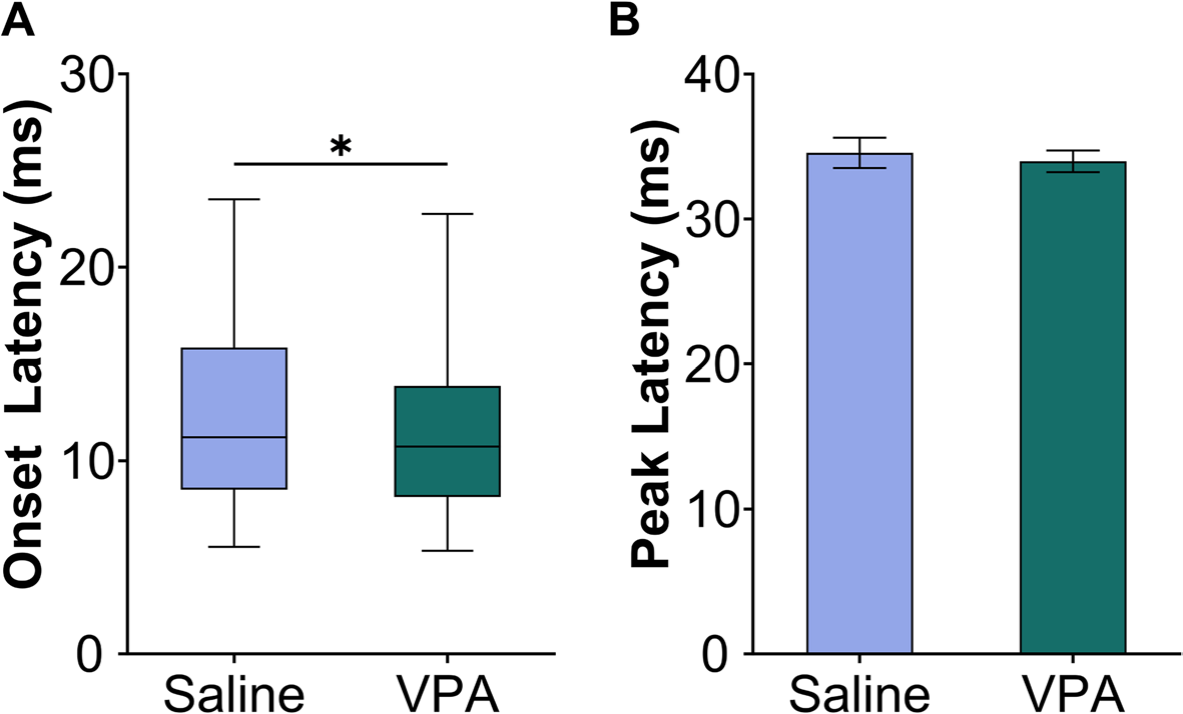
The speech response timing is significantly faster in VPA-exposed rats than saline-exposed rats. **A** Box and whisker plots showing the timing of the onset response latency to speech sounds. The saline-exposed rats were significantly slower than the VPA-exposed rats, indicated with the asterisk. The black line indicates the median, and error bars indicate the 95% confidence interval. **B** Bar plot comparing the timing of the peak response latency to speech sounds. The bars show the mean, and the error bars are 95% confidence intervals

In addition to response strength changes, differences in the timing of responses to speech have been reported in individuals with autism and animal models of ASD [11, 29, 38, 39]. In this study, there was a significant difference in the onset latency, but not in peak latency between the experimental groups. Contrary to the previous findings, the saline-exposed rats had a significantly slower onset response latency to speech sounds than the VPA-exposed rats, while the peak response latency was unaltered between groups (Mann–Whitney *U*, onset: *p* = 0.046; GLMM, Peak: *F*(1,825) = 0.20, *p* = 0.65; Fig. 6, Additional Figure S6). Overall, our results suggest that VPA-exposed rats have altered responses to speech sounds.

### IC responses to tones are unaltered in VPA-exposed rats

**Fig. 7:**
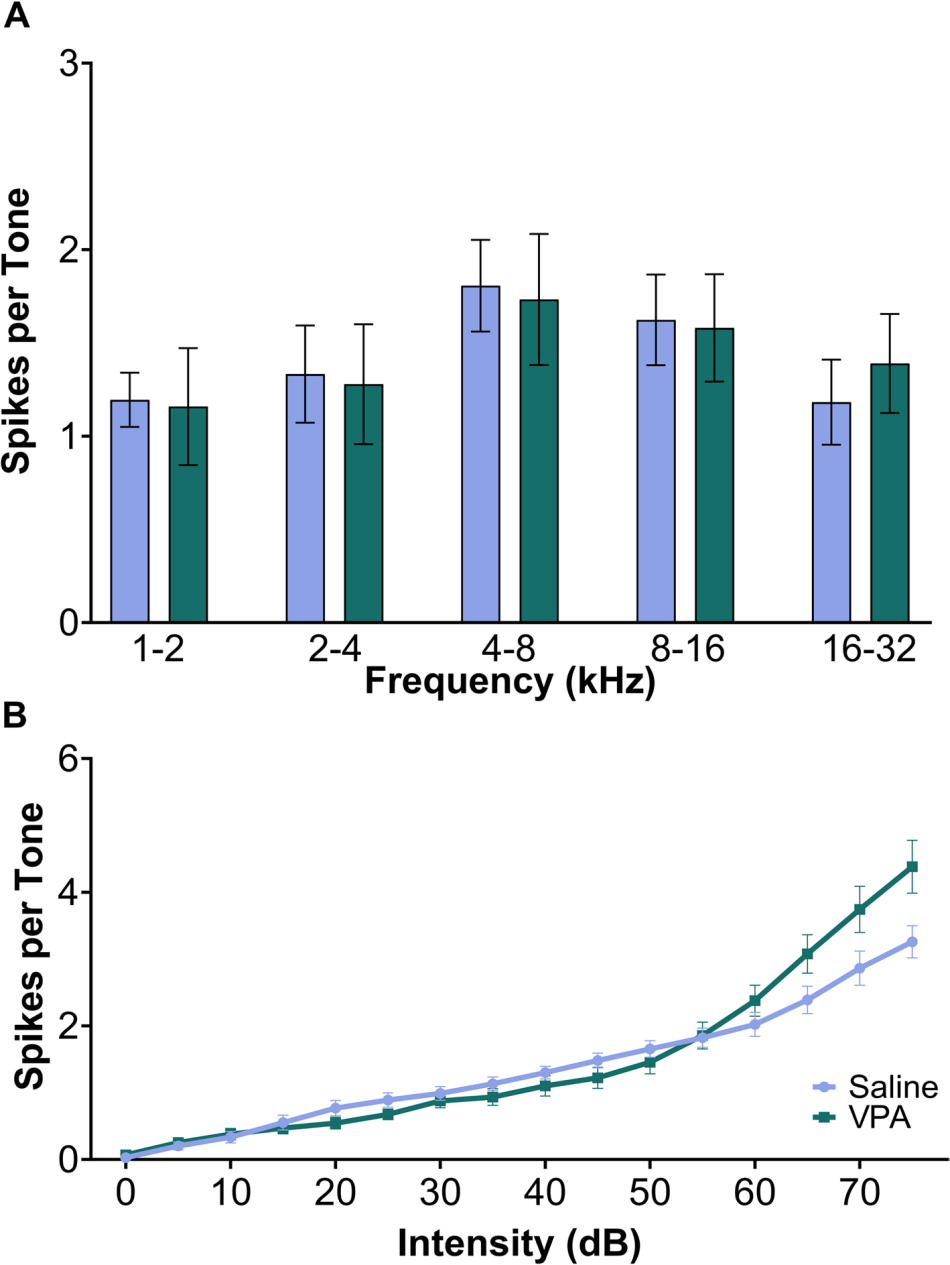
The IC response to pure tones of 1–32 kHz and 0–75 dB were unaltered in VPA-exposed rats. **A** Bar plots showing the average number of spikes across tone frequency binned in 1 octave bins. The error bars are the standard error of the mean across rats. There were no significant differences in the response strength comparing saline-exposed rats to VPA-exposed rats across frequency bins. **B** Rate-intensity function comparing the number of spikes evoked per tone based on sound intensity. The error bars represent the standard error of the mean across rats. For both groups, as the sound intensity increased, there was an increase in the number of evoked spikes. However, no significant differences were found between groups across each intensity

In addition to speech sounds, tones consisting of frequencies from 1 to 32 kHz and intensities from 0 to 75 dB were presented to investigate alterations in tonotopic organization and receptive field properties in the inferior colliculus. To investigate whether the VPA rats exhibit alterations in the response to tones in the IC, the number of spikes tuned to multiple frequency ranges was compared between the two groups. There were no significant differences between VPA-exposed rats and saline-exposed rats (two-way ANOVA, *F*(1,175) = 0.00000204, *p* = 0.999, Fig. 7a). Next, the rate-intensity function to tones was examined to quantify the response to tones at multiple sound intensity levels. As expected, for both groups, increasing tone intensity evoked stronger IC responses (two-way ANOVA, *F*(15,560) = 2.35, *p* = 0.0029). Comparing saline-exposed and VPA-exposed rats, there were no significant differences between experimental groups across intensities (two-way ANOVA, *F*(1,560) = 0.532, *p* = 0.466, Fig. 7b).

Additionally, receptive field properties including response threshold, bandwidths at 10 and 40 dB above the threshold, onset and peak latency, end of peak latency, and spontaneous firing rates were examined between groups. There were no significant differences between the groups for any receptive field properties (GLMM, threshold: *F*(1,35) = 0.15, *p* = 0.70; bandwidth at 10 dB: *F*(1,35) = 0.729, *p* = 0.40; bandwidth at 40 dB: *F*(1,35) = 0.42, *p* = 0.52; onset latency: *F*(1,35) = 0.05, *p* = 0.83; peak latency: *F*(1,35) = 0.25, *p* = 0.62; end of peak latency: *F*(1,35) = 0.47, *p* = 0.50; and spontaneous firing rate: *F*(1,35) = 0.001, *p* = 0.97, Table 1). Overall, VPA-exposed rats did not show significant differences in their IC responses to tones compared to saline-exposed rats.

**Table 1:** Receptive field properties based on responses to tones compared between saline-exposed and VPA-exposed rats

	Saline		VPA	
	Mean	Median (95% CI)	Mean	Median (95% CI)
Threshold (dB)	17.3	18.0 (15.9–25.6)	17.3	15.3 (13.9–23.0)
Bandwidth 10 dB (octaves)	1.3	1.1 (1.0–1.6)	1.5	1.5 (1.1–1.9)
Bandwidth 40 dB (octaves)	1.7	1.6 (1.1–1.9)	1.8	1.8 (1.0–2.4)
Latency (ms)	8.2	7.9 (7.6–8.6)	8.0	8.2 (7.5–9.1)
Peak latency (ms)	13.4	13.3 (12.7–13.9)	12.5	12.9 (11.6–14.3)
End of peak (ms)	27.1	27.3 (26.4–27.6)	25.5	26.4 (24.0–27.0)
Spontaneous (Hz)	30.7	24.5 (8.9–37.4)	29.2	20.5 (12.8–34.5)

All receptive field properties were normally distributed and a generalized linear mixed model was used for comparison. Overall, no significant differences were observed between groups

### IC temporal processing is intact in VPA-exposed rats

**Fig. 8:**
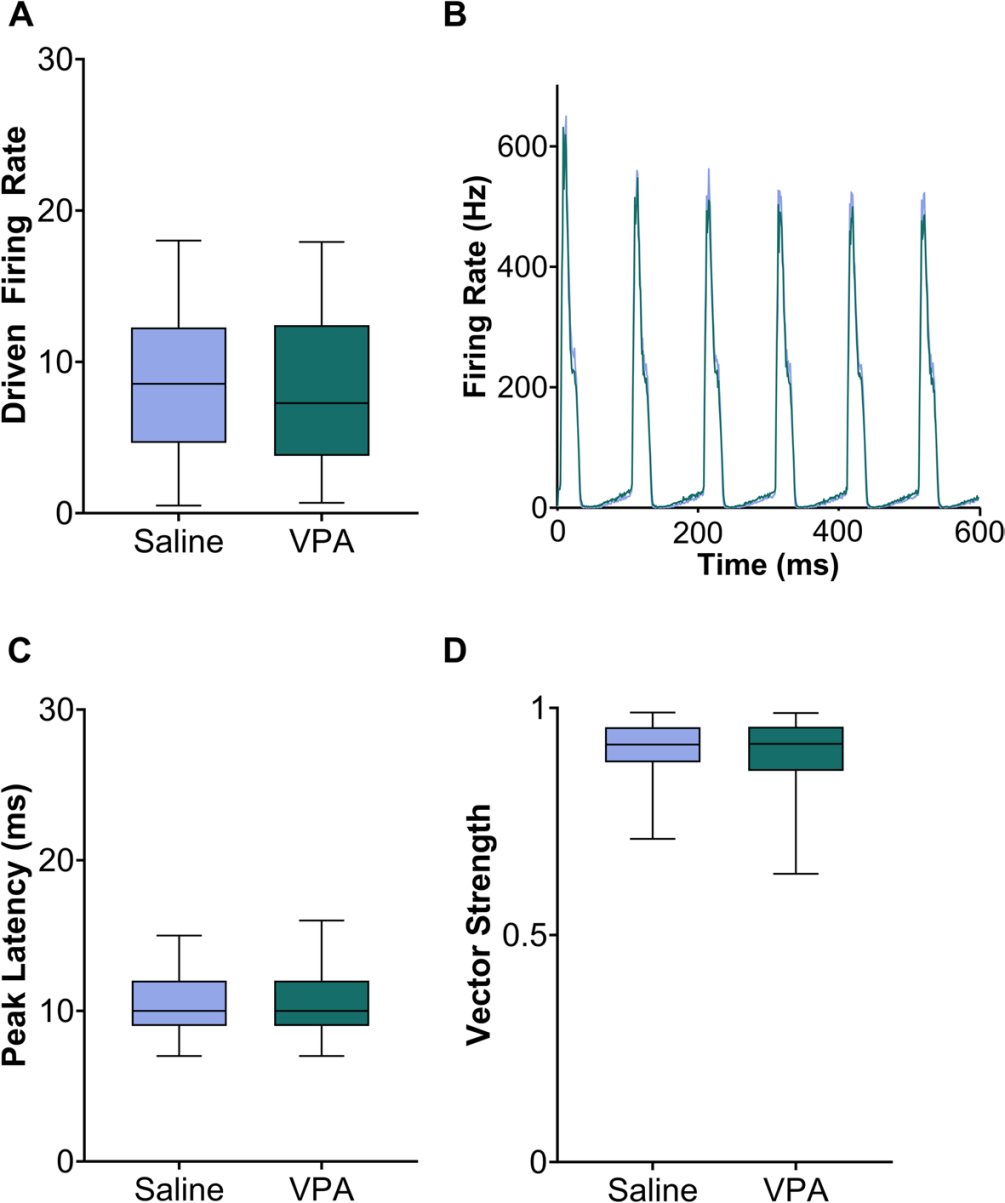
Responses to 10 Hz trains of noise bursts were compared between the groups. **A** The response strength evoked by noise burst trains showed no alterations in VPA-exposed rats. The black line indicates the median, and error bars indicate the 95% confidence interval. **B** Average peristimulus time histogram (PSTH) to noise bursts presented six times at 10 Hz. **C** Peak latency response to the first noise burst in saline-exposed and VPA-exposed rats. No significant differences were observed between groups. **D** The vector strength was unaltered in VPA-exposed rats compared to saline-exposed rats

In both human and animal models of autism, the cortical phase locking to rapidly presented sounds is often impaired, with both a decreased neural amplitude and less synchronous responses evoked by sounds [1, 11, 39, 40]. To investigate whether this is also observed in the inferior colliculus, we presented 10 Hz trains of noise bursts. As seen with responses to tones, there was no significant difference in the peak firing rate evoked by noise burst trains between the saline-exposed rats and the VPA-exposed rats (Mann–Whitney *U*, *p* = 0.18; Fig. 8a, b, Additional Figure S7a, b). In addition, the timing of the first peak of the response was not significantly different between the groups (Mann–Whitney *U*, *p* = 0.55; Fig. 8c, Additional Figure S7c). To quantify synchrony, the vector strength was examined. The vector strength was also unaltered between the two groups (Mann–Whitney *U*, *p* = 0.70; Fig. 8d, Additional Figure S7d). These results suggest that, in the IC, exposure to VPA does not influence temporal processing, at a relatively slow rate of 10 Hz.

### IC neural discrimination accuracy is degraded in VPA-exposed rats

A nearest-neighbor classifier was used to quantify the neural discrimination of pairs of speech sounds. A previous study found that in the anterior auditory field, VPA-exposed rats had significantly worse discrimination accuracy of consonant pairs, but not vowel pairs, compared to saline-exposed rats [11]. In our current study, the discriminability of IC responses to pairs of speech sounds was compared. Overall, the IC neurons of the VPA-exposed rats were significantly less accurate at discriminating stop consonant pairs (D-G, D-T, G-T) compared to saline-exposed rats (Mann–Whitney *U*, *p* = 0.013; Table 2). No significant differences were found between experimental groups in the neural discrimination of stop consonants within either low or high-frequency bins (Mann–Whitney U, low: *p* = 0.09; high: *p* = 0.08). We next examined the affricates and the fricative sounds. For both, the VPA-exposed rats were overall worse at discriminating affricate and fricative pairs of sounds (Mann–Whitney *U*, affricates: *p* = 0.0009; fricatives: *p* = 0.0001), and especially at low-frequency recording sites (Mann–Whitney *U*, affricates: *p* = 0.0038; fricatives: *p* < 0.001). Affricate and fricative sound pair discrimination at high-frequency recordings sites were not significantly different between groups (Mann–Whitney *U*, affricates: *p* = 0.080, fricatives: *p* = 0.114). Comparing neural discrimination to vowel pairs, VPA-exposed rats were significantly worse than saline-exposed rats overall (Mann–Whitney *U*, *p* = 0.026). However, no significant differences were seen between groups at either low-frequency sites or high-frequency sites. These results indicate that neural responses to unique speech sounds evoke more similar neural activity patterns in VPA-exposed rats and more distinct neural activity patterns in saline-exposed rats.

**Table 2:** Neural classifier accuracy based on both the consonant portion of the sound (40 1-ms bins), organized into three different phonetic groups, and the vowel portion of the sound (1 300-ms bin)

		Low frequency (1–8 kHz)		High frequency (9–32 kHz)	
		Saline	VPA	Saline	VPA
Consonant	**Stops** **(D/G/T)**	82.5 (80**–**85)	77.5 (75**–**82.5)	80 (75**–**82.5)	77.5 (75**–**82.5)
	**Affricates** **(Ch/J)**	**95** **(92.5–97.5)**	92.5 (90**–**95)	95 (92.5**–**97.5)	92.5 (90**–**95)
	**Fricatives** **(F/H/S/Sh)**	**97.5** **(97.5–97.5)**	95 (92.5**–**95)	97.5 (95**–**97.5)	95 (95**–**97.5)
Vowels	**(-ad/-eed/-ood)**	82.5 (80–87.5)	77.5 (75**–**82.5)	81.25 (77.5**–**85)	77.5 (75**–**80)

The numbers represented in this table are shown as median (95% confidence interval). The bolded numbers indicate experimental groups that are statistically significant from each other across low and high-frequency recording sites using a Mann–Whitney *U* test. Classifier accuracy was determined for speech sound pairs for low-frequency neurons (1**–**8 kHz) and high-frequency neurons (9**–**32 kHz)

## Discussion

Individuals with ASD have auditory brainstem response (ABR) abnormalities, which present as delayed and weaker brainstem responses to sounds compared to neurotypical individuals [39, 40]. Previous studies have observed anatomical differences along the auditory pathway in rodents prenatally exposed to VPA [11, 13, 15, 40, 41]. The purpose of this study was to investigate subcortical auditory processing physiology in prenatally exposed VPA rats. This study expands on previous studies that have documented that prenatal exposure to VPA affects auditory processing in both cortical and subcortical regions [1, 13, 42]. In this study, VPA-exposed rats had significantly weaker and delayed responses to speech sounds compared to saline-exposed control rats in the inferior colliculus. Meanwhile, VPA-exposed rats had no alterations in the response to tones and noise bursts compared to saline-exposed control rats. These results suggest that VPA-exposed rats have intact IC processing of simple sounds but are impaired at processing complex stimuli.

### Complex versus simple stimuli processing in ASD

Children with ASD have difficulty processing spectrotemporally complex sounds, like speech sounds, to a greater extent than simple sounds, like tones [1, 38, 43–45]. These children have significantly weaker and delayed responses to complex sounds than they do simple sounds when compared to typically developing children [1, 37, 40, 44, 46–49]. For example, one study found that when children with autism were presented with the speech sound /da/, these children had significantly weaker ABR responses compared to typically developing children [50]. Our current study supports the previous findings; the VPA-exposed rats displayed significantly degraded IC responses to speech sounds compared to saline-exposed rats, but their responses to tones were intact. Previously, VPA-rats were found to also process speech sounds significantly weaker and slower in the anterior auditory field (AAF) compared to saline-exposed rats [42]. In addition to the abnormal cortical and subcortical auditory processing observed in these rats, VPA rats also perform consonant discrimination tasks, but not vowel discrimination tasks, with significantly less accuracy than the saline-exposed control rats [10]. These findings together provide further evidence that VPA exposure affects the processing of complex sound stimuli. This disrupted auditory processing could potentially contribute to the receptive language and social communication impairments that are commonly observed in children with ASD [9, 43].

### The auditory pathway in VPA-exposed rodents

Previous studies have found that VPA-exposed rats have degraded processing of sounds in the primary auditory cortex and the anterior auditory field (AAF) [11, 12]. While no studies have documented neuron spiking activity subcortically in VPA-exposed rodents, multiple papers document that there is abnormal neural structure subcortically in VPA-exposed rodents. VPA-exposed rodents have fewer neurons in the ventral cochlear nucleus, superior olivary complex, inferior colliculus, and medial geniculate nucleus of the thalamus [16, 17]. Interestingly, a recent paper exhibited elevated ABR thresholds and increased latencies to clicks in VPA-exposed animals at 22 days of age, but not 60 days of age [15]. This fascinating finding suggests that there may be a delayed maturation of auditory brainstem responses to simple sounds, like clicks, and provides hope that the severely impacted responses observed in response to more complex sounds, like speech, could potentially improve with age.

### The auditory pathway in children with ASD

Previous studies have found alterations in the neural response to sounds across the auditory pathway in individuals with ASD. Subcortically, individuals with ASD have been documented to have normal click-evoked brainstem responses, but neural synchrony and phase-locking brainstem response deficits to speech sounds [39, 40]. Responses in the primary auditory cortex were significantly delayed in children with both ASD and hypersensitivity compared to children with ASD without hypersensitivity and typically developing children [51]. Interestingly, another study has documented unaltered speech-evoked responses in the primary auditory cortex but reduced speech-evoked responses in the superior temporal gyrus (STG) in individuals with ASD [52]. Response latencies are also greatly degraded in individuals with ASD in the STG. Compared to typically developing children, verbal children with ASD have significantly longer STG latencies to vowel sounds, language-impaired children with ASD have latencies that are further delayed, and minimally verbal/non-verbal children with ASD have profoundly delayed latencies to vowel sounds in the STG [3]. Overall, children with ASD often exhibit degraded neural responses to speech sounds across the auditory pathway.

## Future studies

The current study showed that VPA-exposed rats have degraded responses to speech sounds in the inferior colliculus, specifically to stop consonants and vowels. The neural classifier also revealed that VPA-exposed rats are worse at discriminating consonants and vowels compared to saline-exposed rats. This finding is consistent with previous studies, as Engineer et al. (2014) found that VPA-exposed rats performed worse at consonant discrimination tasks compared to saline-exposed rats. Extensive auditory discrimination training improved the responses in the anterior auditory field in trained VPA-exposed rats compared with untrained VPA-exposed rats. Therefore, it may also be beneficial to document inferior colliculus responses after extensive auditory discrimination training.

Histological verification of electrode placement and recording sites was not obtained in this study [53]. Instead, consistent with previous studies, the orderly tonotopy observed in the CNIC was used to identify the recording location [22, 24, 27, 53]. Our observation that the CNIC tonotopic axis length is significantly decreased is consistent with multiple previous studies that have documented that prenatal VPA exposure alters midbrain size [34, 35]. Future studies with electrolytic lesions will be necessary to examine the differences observed in VPA-exposed rats in multiple regions of the IC.

Future studies are also needed in additional models of ASD. Rodents who are prenatally exposed to valproic acid model fetal valproate syndrome, which is observed in children who were prenatally exposed to valproic acid (sodium valproate). Prenatal valproate exposure is associated with an increased risk of ASD, however, it represents a small fraction of the etiologies associated with ASD. Exploring the similarities and differences across a range of ASD models may provide greater insight into the contribution of auditory processing to speech and communication deficits [4, 54, 55].

Furthermore, the usage of vagus nerve stimulation (VNS) paired with behavioral training may improve auditory processing in both animal models and children with autism [56, 57]. Previous studies have found that vagus nerve stimulation significantly improves the frequency and severity of seizures, which are common co-morbid disorders in children with autism [58–62]. Additionally, pairing VNS with sounds significantly strengthens responses in typically hearing rats across the auditory pathway [24, 63]. VNS has also been paired with tones in a rat autism model of Rett syndrome, heterozygous *Mecp2* rats [29]. Pairing VNS with tones for 20 days significantly improved the primary auditory cortex response to sounds in *Mecp2* rats. Future studies pairing VNS with sounds are needed to document both behavioral discrimination ability and auditory responses along the auditory pathway in multiple rat models of ASD.

### Supplementary Information

**Additional file 1.** Post-stimulus time histogram showing the IC response to the 15 different speech sounds that were presented during recording. The sound waveform is plotted behind the PSTH in gray.

**Additional file 2.** Violin plot showing the number of driven spikes evoked at each IC recording site for each speech sound. The driven rate was quantified using the 400 ms duration of the response. The dashed line indicates the median, and the dotted lines indicate the quartiles. The asterisks indicate experimental groups that are statistically significant from each other using a Mann–Whitney U test. The symbols represent the following: *** *p* < 0.001.

**Additional file 3.** A) Left, violin plots showing the number of driven spikes evoked at each IC recording site for the consonant portion of each stop consonant sound. The driven rate was quantified using the 40 ms response to the consonant portion of the sounds. The dashed line indicates the median, and the dotted lines indicate the quartiles. The asterisk indicates experimental groups that are statistically significant from each other using a Mann–Whitney U test. Right, violin plots depicting the average group response strength evoked by the consonants ‘d’, ‘g’, and ‘t’ presented at 60 dB. B) Left, violin plots showing the number of driven spikes evoked at each IC recording site for the consonant portion of each affricate sound. Right, violin plots depicting the average group response strength evoked by the consonants ‘ch’, and ‘j’ presented at 60 dB. C) Left, violin plots showing the number of driven spikes evoked at each IC recording site for the consonant portion of each fricative sound. The driven rate was quantified using the 40 ms response to the consonant portion of the sounds. Right, violin plots depicting the average group response strength evoked by the consonants ‘f’, ‘h’, ‘s’, and ‘sh’ presented at 60 dB.

**Additional file 4.** A) Violin plots showing the number of driven spikes evoked at each IC recording site for the vowel portion of each speech sound. The driven rate was quantified using the 300 ms response to the vowel portion of the sounds. The dashed line indicates the median, and the dotted lines indicate the quartiles. The asterisk indicates experimental groups that are statistically significant from each other using a Mann–Whitney U test. B) Violin plots depicting the average group response strength evoked by the vowels ‘a’, ‘ee’, and ‘oo’ presented at 60 dB.

**Additional file 5.** A) Violin plots depicting the average group response strength evoked by the sound ‘dad’ presented at 75, 60, and 45 dB. The driven rate was quantified using the 400 ms response to the sounds. The dashed line indicates the median, and the dotted lines indicate the quartiles. The asterisk indicates experimental groups that are statistically significant from each other using a Mann–Whitney U test. All asterisks are represented in APA style: **p < 0.005, *p < 0.05. B) Violin plots depicting the average group response strength evoked by the sound ‘shad’ presented at 75, 60, and 45 dB.

**Additional file 6.** A) Violin plots showing the timing of the onset response latency to speech sounds. The saline-exposed rats were significantly slower than the VPA-exposed rats, indicated with the asterisk. The dashed line indicates the median, and the dotted lines indicate the quartiles. B) Bar plot comparing the timing of the peak response latency to speech sounds. The bars show the mean, and the error bars are 95% confidence intervals.

**Additional file 7.** A) Violin plots showing the number of driven spikes evoked at each IC recording site for the noise bursts. The dashed line indicates the median, and the dotted lines indicate the quartiles. B) Average peristimulus time histogram (PSTH) to noise bursts presented six times at 10 Hz. C) Violin plots depicting the peak latency to the first noise burst in saline-exposed and VPA-exposed rats. The dashed line indicates the median, and the dotted lines indicate the quartiles. D) Violin plots comparing the vector strength to noise bursts. The dashed line indicates the median, and the dotted lines indicate the quartiles.

## Data Availability

All datasets and analysis codes are available upon request to the corresponding author.

## Abbreviations

ASD: Autism spectrum disorders
VPA: Valproic acid
IC: Inferior colliculus
AAF: Anterior auditory field
STG: Superior temporal gyrus
VNS: Vagus nerve stimulation
