# Supplementary material for: Degraded inferior colliculus responses to complex sounds in prenatally exposed VPA rats
Source: J Neurodev Disord. 2024 Jan 2;16:2. doi: 10.1186/s11689-023-09514-9 (PMC10759431; doi:10.1186/s11689-023-09514-9)
Supplement: Supplementary file 1 — Additional file 1. Post-stimulus time histogram showing the IC response to the 15 different speech sounds that were presented during recording. The sound waveform is plotted behind the PSTH in gray. [file 11689_2023_9514_MOESM1_ESM.pdf]

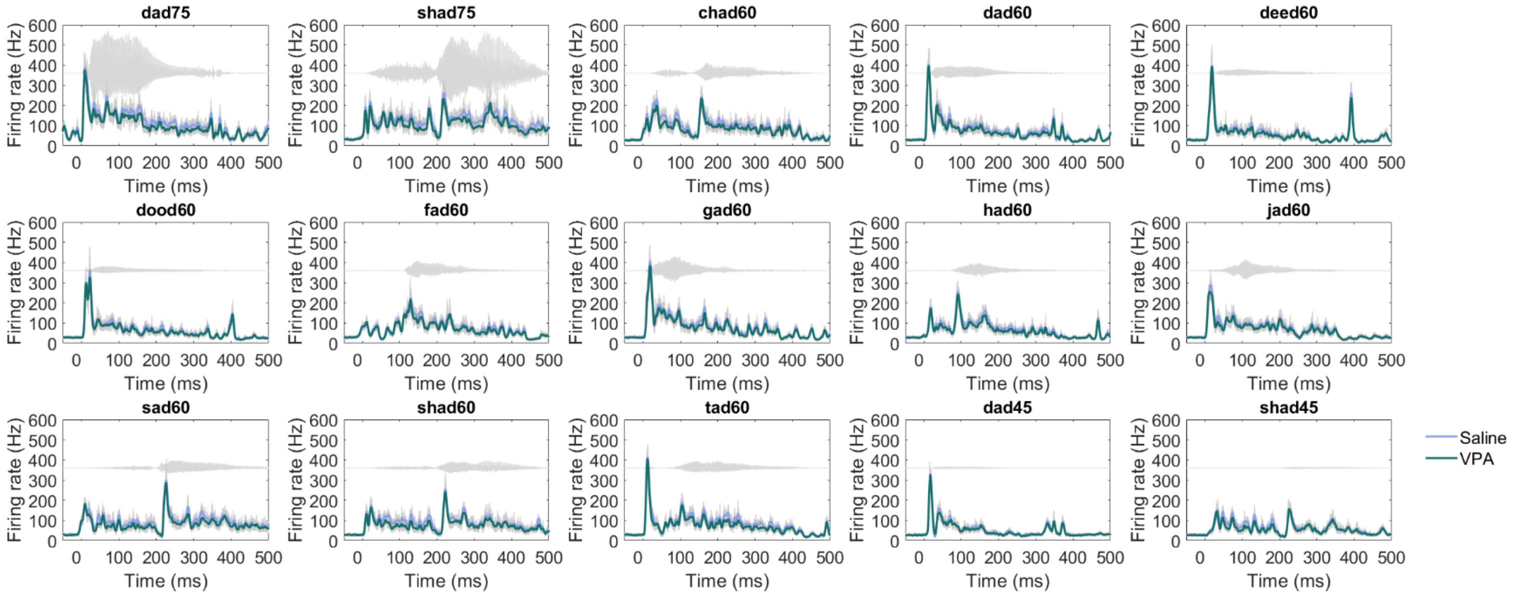

Additional Files 1 Post-stimulus time histogram (PSTH) showing the IC responses to the 15 different speech sounds that were presented during recording. The title of each PSTH contains the speech sound with the intensity (in dB). The sound waveform is plotted behind the PSTH in gray.
