# Supplementary material for: Degraded inferior colliculus responses to complex sounds in prenatally exposed VPA rats
Source: J Neurodev Disord. 2024 Jan 2;16:2. doi: 10.1186/s11689-023-09514-9 (PMC10759431; doi:10.1186/s11689-023-09514-9)
Supplement: Supplementary file 2 — Additional file 2. Violin plot showing the number of driven spikes evoked at each IC recording site for each speech sound. The driven rate was quantified using the 400 ms duration of the response. The dashed line indicates the median, and the dotted lines indicate the quartiles. The asterisks indicate experimental groups that are statistically significant from each other using a Mann–Whitney U test. The symbols represent the following: *** p < 0.001. [file 11689_2023_9514_MOESM2_ESM.pdf]

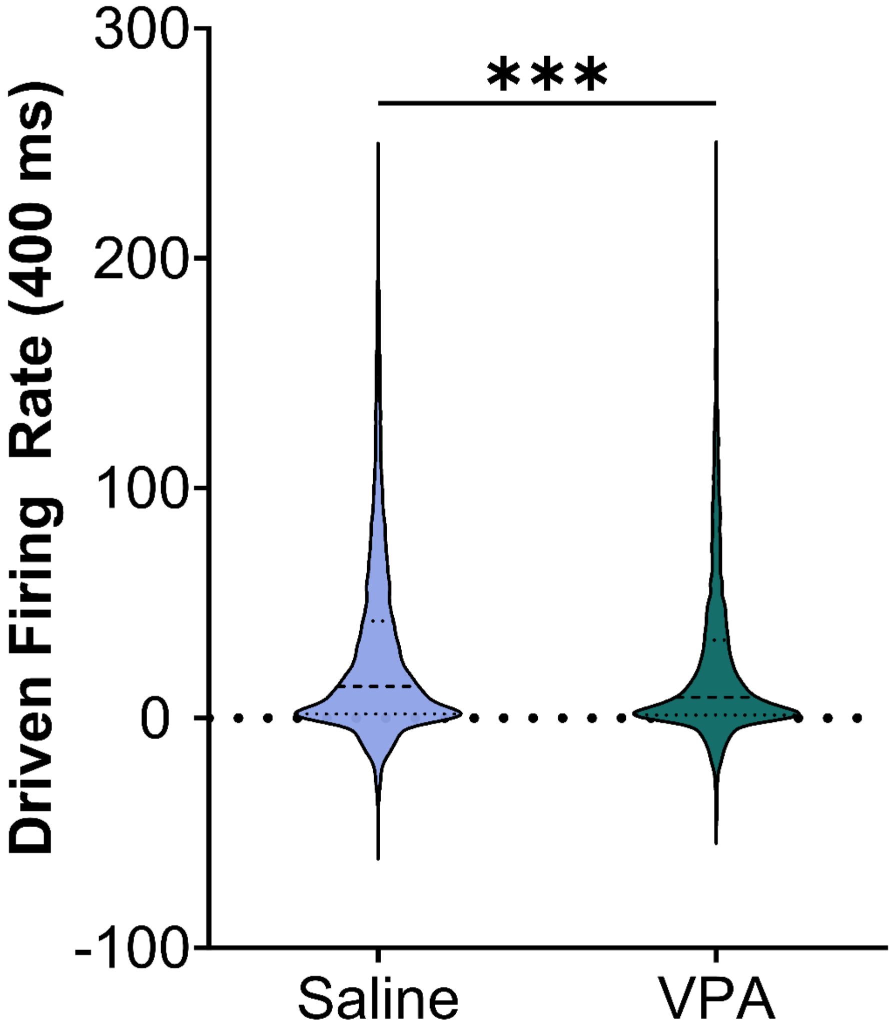


**Additional File 2** Violin plot showing the number of driven spikes evoked at each IC recording site for each speech sound. The driven rate was quantified using the 400 ms duration of the response. The dashed line indicates the median, and the dotted lines indicate the quartiles. The asterisks indicate experimental groups that are statistically significant from each other using a Mann-Whitney U test. The symbols represent the following: *** $p<0.001$.
