# Supplementary material for: Degraded inferior colliculus responses to complex sounds in prenatally exposed VPA rats
Source: J Neurodev Disord. 2024 Jan 2;16:2. doi: 10.1186/s11689-023-09514-9 (PMC10759431; doi:10.1186/s11689-023-09514-9)
Supplement: Supplementary file 4 — Additional file 4. A) Violin plots showing the number of driven spikes evoked at each IC recording site for the vowel portion of each speech sound. The driven rate was quantified using the 300 ms response to the vowel portion of the sounds. The dashed line indicates the median, and the dotted lines indicate the quartiles. The asterisk indicates experimental groups that are statistically significant from each other using a Mann–Whitney U test. B) Violin plots depicting the average group response strength evoked by the vowels ‘a’, ‘ee’, and ‘oo’ presented at 60 dB. [file 11689_2023_9514_MOESM4_ESM.pdf]

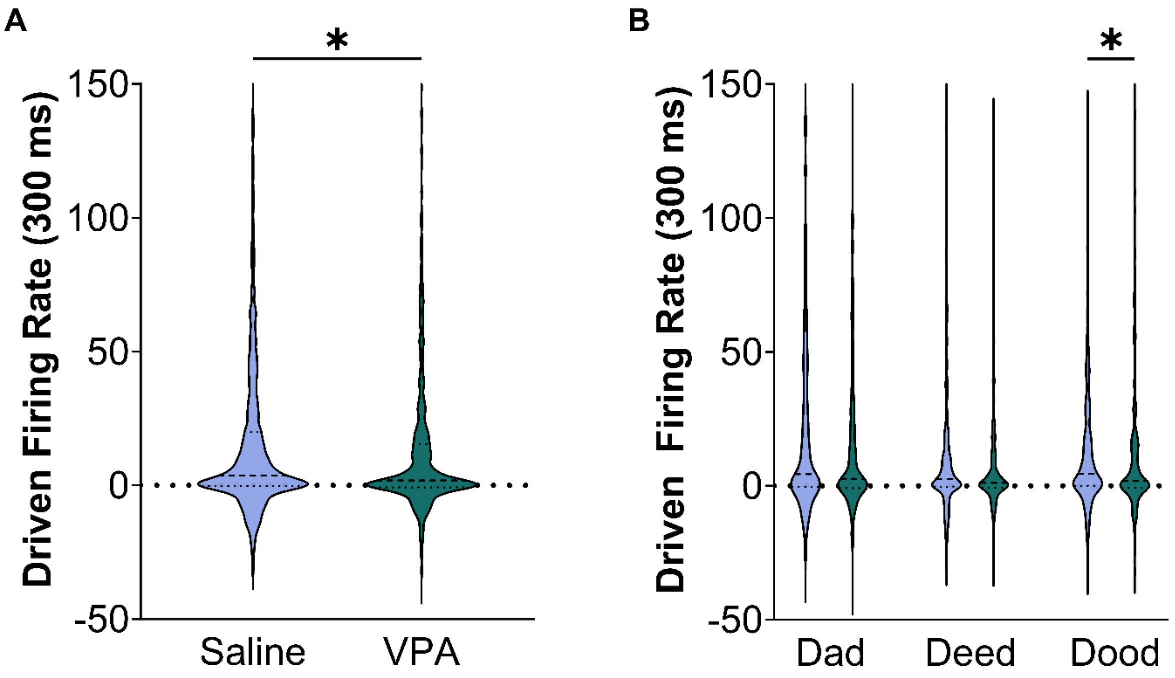

Additional File 4 A) Violin plots showing the number of driven spikes evoked at each IC recording site for the vowel portion of each speech sound. The driven rate was quantified using the 300 ms response to the vowel portion of the sounds. The dashed line indicates the median, and the dotted lines indicate the quartiles. The asterisk indicates experimental groups that are statistically significant from each other using a Mann-Whitney U test, p=0.01. **B)** Violin plots depicting the average group response strength evoked by the vowels ‘a’, ‘ee’, and ‘oo’ presented at 60 dB.
