# Supplementary material for: Degraded inferior colliculus responses to complex sounds in prenatally exposed VPA rats
Source: J Neurodev Disord. 2024 Jan 2;16:2. doi: 10.1186/s11689-023-09514-9 (PMC10759431; doi:10.1186/s11689-023-09514-9)
Supplement: Supplementary file 5 — Additional file 5. A) Violin plots depicting the average group response strength evoked by the sound ‘dad’ presented at 75, 60, and 45 dB. The driven rate was quantified using the 400 ms response to the sounds. The dashed line indicates the median, and the dotted lines indicate the quartiles. The asterisk indicates experimental groups that are statistically significant from each other using a Mann–Whitney U test. All asterisks are represented in APA style: **p < 0.005, *p < 0.05. B) Violin plots depicting the average group response strength evoked by the sound ‘shad’ presented at 75, 60, and 45 dB. [file 11689_2023_9514_MOESM5_ESM.pdf]

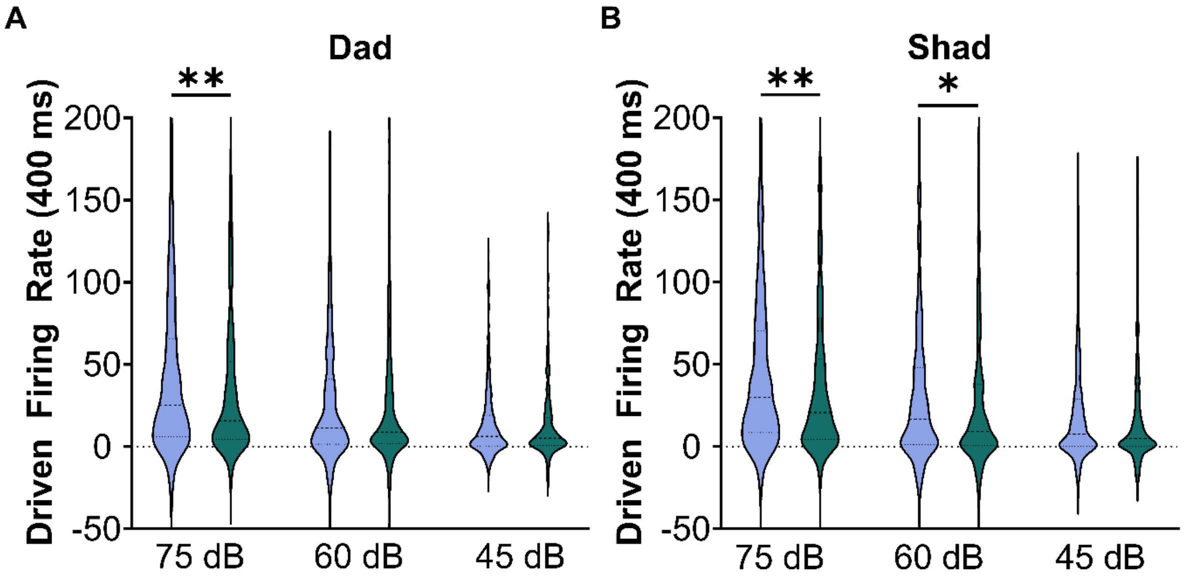

Additional File 5 A) Violin plots depicting the average group response strength evoked by the sound 'dad' presented at 75, 60, and 45 dB. The driven rate was quantified using the 400 ms response to the sounds. The dashed line indicates the median, and the dotted lines indicate the quartiles. The asterisk indicates experimental groups that are statistically significant from each other using a Mann-Whitney U test, p<0.01. **B)** Violin plots depicting the average group response strength evoked by the sound 'shad' presented at 75, 60, and 45 dB. The asterisks indicate experimental groups that are statistically significant from each other using a Mann-Whitney U test. All asterisks are represented in APA style: **p<0.01, *p<0.05.
