# Supplementary material for: Degraded inferior colliculus responses to complex sounds in prenatally exposed VPA rats
Source: J Neurodev Disord. 2024 Jan 2;16:2. doi: 10.1186/s11689-023-09514-9 (PMC10759431; doi:10.1186/s11689-023-09514-9)
Supplement: Supplementary file 6 — Additional file 6. A) Violin plots showing the timing of the onset response latency to speech sounds. The saline-exposed rats were significantly slower than the VPA-exposed rats, indicated with the asterisk. The dashed line indicates the median, and the dotted lines indicate the quartiles. B) Bar plot comparing the timing of the peak response latency to speech sounds. The bars show the mean, and the error bars are 95% confidence intervals. [file 11689_2023_9514_MOESM6_ESM.pdf]

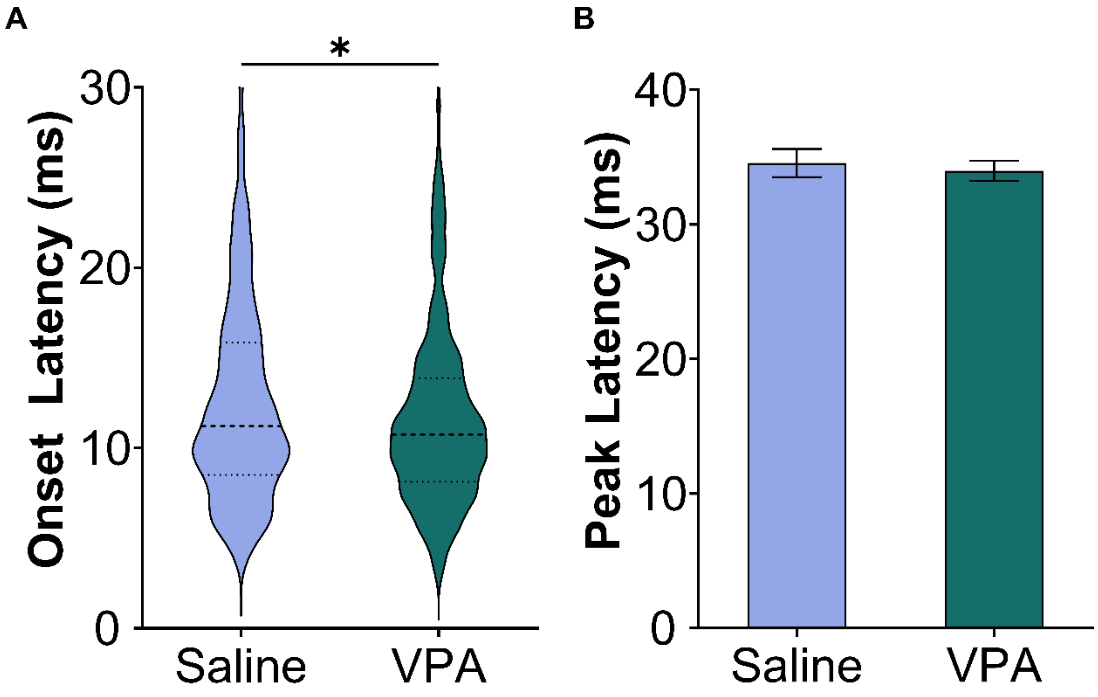

Additional File 6 A) Violin plots showing the timing of the onset response latency to speech sounds. The saline-exposed rats were significantly slower than the VPA-exposed rats, indicated with the asterisk, which represents p<0.05. The dashed line indicates the median, and the dotted lines indicate the quartiles. **B)** Bar plot comparing the timing of the peak response latency to speech sounds. The bars show the mean, and the error bars are 95% confidence intervals.
