# Supplementary material for: Degraded inferior colliculus responses to complex sounds in prenatally exposed VPA rats
Source: J Neurodev Disord. 2024 Jan 2;16:2. doi: 10.1186/s11689-023-09514-9 (PMC10759431; doi:10.1186/s11689-023-09514-9)
Supplement: Supplementary file 7 — Additional file 7. A) Violin plots showing the number of driven spikes evoked at each IC recording site for the noise bursts. The dashed line indicates the median, and the dotted lines indicate the quartiles. B) Average peristimulus time histogram (PSTH) to noise bursts presented six times at 10 Hz. C) Violin plots depicting the peak latency to the first noise burst in saline-exposed and VPA-exposed rats. The dashed line indicates the median, and the dotted lines indicate the quartiles. D) Violin plots comparing the vector strength to noise bursts. The dashed line indicates the median, and the dotted lines indicate the quartiles. [file 11689_2023_9514_MOESM7_ESM.pdf]

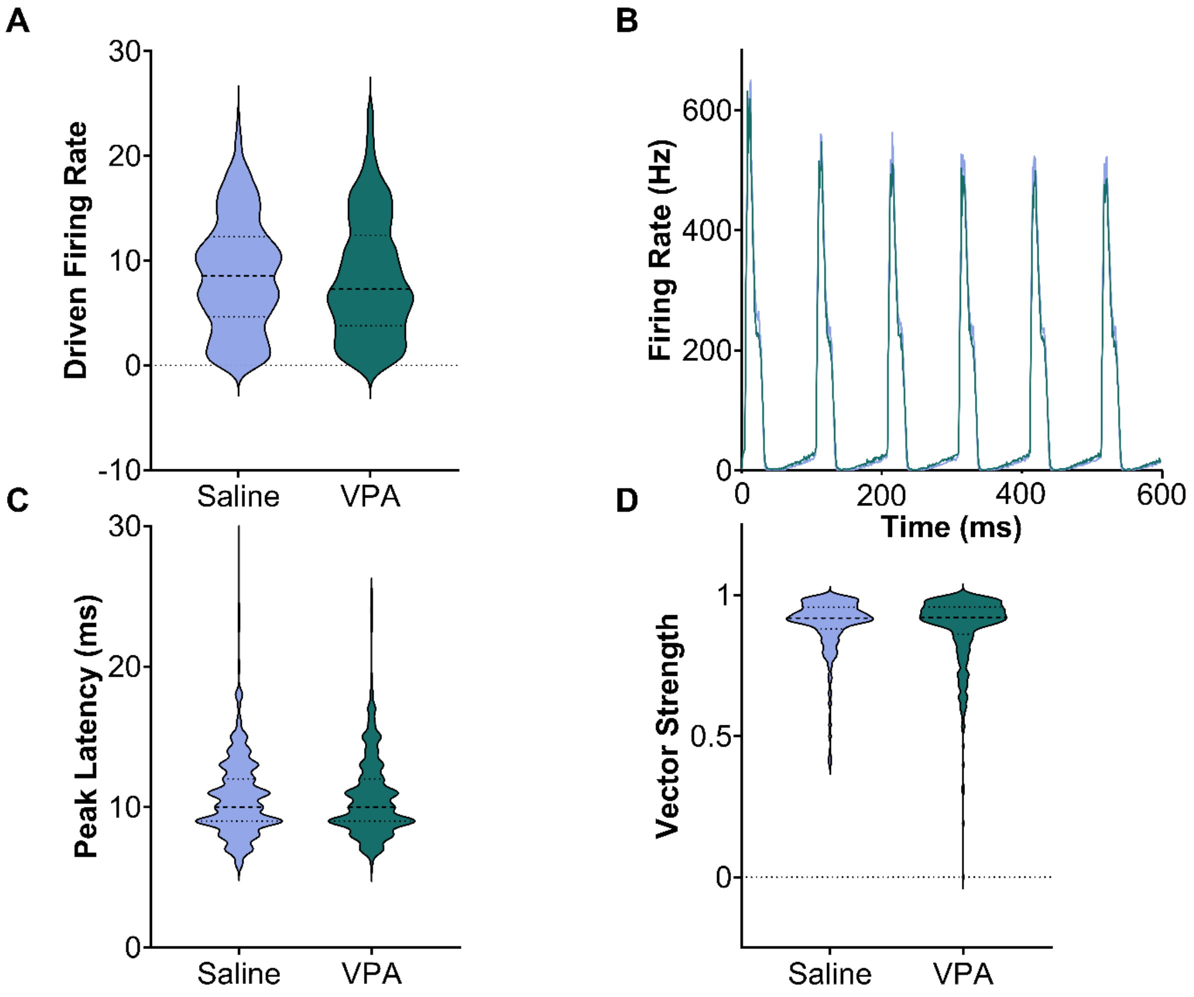

Additional File 7 A) Violin plots showing the number of driven spikes evoked at each IC recording site for the noise bursts. The dashed line indicates the median, and the dotted lines indicate the quartiles. **B)** Average peristimulus time histogram (PSTH) to noise bursts presented six times at 10 Hz. **C)** Violin plots depicting the peak latency to the first noise burst in saline-exposed and VPA-exposed rats. The dashed line indicates the median, and the dotted lines indicate the quartiles. **D)** Violin plots comparing the vector strength to noise bursts. The dashed line indicates the median, and the dotted lines indicate the quartiles.
